# Index Medicus

**Published:** 1895-01

**Authors:** 


INDEX MEDICUS. A monthly journal devoted to the cataloguing the titles of all medical articles published. Editors, Dr. John S. Billings, Surgeon U. S. Army, and Dr. Robert Fletcher, M. R. C. S., England. Subscription price, $10.00 a year, payable in advance. George S. Davis, Publisher, Detroit, Mich., U. S. A.

The scope and purpose of the journal, now in the sixteenth year of its career, are well known to the prominent members of the medical profession at home and abroad; but even to them, as well as to those who may desire to become subscribers, a statement of the method of its production may be of interest.

The *Index Medicus* is a royal octavo journal, each number containing 48 double-column pages, handsomely printed from clear type on superior quality of paper—especial care being taken to secure typographical accuracy and perfect finish.

The library of the Surgeon-General's office in Washington, one of the most important medical libraries in existence, obtains, by subscription or exchange, all the medical journals and transactions of medical societies published in any language in any part of the world. It has required years of diligent inquiry and research to ascertain the names, merely, of these publications and to perfect the system by which they are obtained, and it is safe to say there is no such complete collection of current medical literature to be found elsewhere. As fast as these journals are received the titles of all original articles are copied to form part of the Index Catalogue now being issued under the authority of Congress.

This extensive work, of which the fifteenth volume (extending through the letter U) is now completed, is a catalogue not only of authors, but of all subjects of medicine, the latter being arranged under a very comprehensive classification. For example, the first six volumes of the work contain under these subject-headings 64,142 books and 219,154 journal articles. This is in addition to the author-titles, which amount to 58,886.

All the works thus indexed are to be found in the library, and are therefore available for consultation.

The *Index Medicus* comprises, in addition to the titles copied from the daily indexing of the current literature received at the library of the Surgeon-General's office, those of all new medical works, or new editions, which may not form part of that collection; it is therefore, as nearly as possible, *a complete bibliography of medicine.* The value of such a record, the only one of its kind in existence, can hardly be over-rated; and as the edition is small and not stereotyped the volumes are certain to become scarce and enhanced in price.

The *Index Medicus* has heretofore been published at a very considerable pecuniary loss. Many suggestions from those interested in the welfare of the enterprise have been received by the publisher to the effect that a material reduction in the subscription price would, by making the work popular and its possession by the mass of the profession possible, establish it on a permanently self-sustaining basis. In view of these facts the publisher begs to announce that, while the loss resulting from the continuance of the enterprise at the present time, which must necessarily be assumed by him, will not admit of a reduction in the subscription price at once, he proposes to reduce the price as soon as the support received from the medical profession will warrant such action. It is thus to the advantage of every subscriber to use his influence in behalf of the *Index Medicus* in the circle of his professional friends, and it is hoped the requisite support may be speedily secured.

